# New players in the preventive treatment of migraine

**DOI:** 10.1186/s12916-015-0522-1

**Published:** 2015-11-10

**Authors:** Dimos D. Mitsikostas, Alan M. Rapoport

**Affiliations:** Neurology Department, Athens Naval Hospital, 70 Dinokratous Street, 11521 Athens, Greece; The David Geffen School of Medicine at UCLA, Los Angeles, CA 90095 USA; 4255 Jefferson Avenue, Suite 27, Woodside, California, CA 94062 USA

**Keywords:** Migraine, Cluster headache, Prevention, CGRP, Monoclonal antibodies, Neurostimulation, Neuromodulation, Vagus nerve stimulation, Supraorbital and supratrochlear nerve stimulation

## Abstract

Migraine is a common, chronic disorder of the brain causing much disability, as well as personal, familial and societal impact. Several oral preventive agents are available in different countries for the prevention of migraine, but none have performed better than 50 % improvement in 50 % of patients in a clinical trial. Additionally, each has various possible adverse events making their tolerability less than optimal. Recently, three monoclonal antibodies targeting the calcitonin gene-related peptide (CGRP) ligand (LY2951742, ALD403 and TEV-48125) and one targeting the CGRP receptor (AMG 334) have completed phase 2 trials, and the results have been reported. These early results show them all to be somewhat more effective than placebo, with no serious adverse events. Three have been studied for episodic migraine, and only TEV-48125 has been studied for both high frequency episodic and chronic migraine. Moreover, preliminary data suggests that neurostimulation is effective in migraine treatment, including stimulation of the sphenopalatine ganglion, transcutaneous supraorbital and supratrochlear nerve, and transcutaneous vagus nerve. In this article, these innovative therapies will be reviewed.

## Background

Migraine is a common, chronic neurovascular disorder of the brain with cranial autonomic findings. It is characterized by recurrent, severe attacks of headaches often associated with other symptoms and much disability, as well as personal, familial and societal impact. It affects approximately 12 % of the general population in Western countries, and affects three times more women than men [1]. Migraine disability is related to the frequency and severity of attacks together with the number and type of existing comorbidities. Mood disorders, obesity and medication overuse are the most common co-occurring chronic disorders that significantly amplify the impact of migraine on the individual [2, 3].

Migraine attacks are episodic and average 1–3 times per month in most migraineurs. Chronic migraine is defined as headaches on at least 15 days per month for at least 3 months in patients with a history of episodic migraine. Additionally, patients must present features of migraine on at least 8 days per month. Up to 30 % of migraineurs have an aura preceding or coexisting with the headache, which is usually a visual aberration lasting about 20 minutes, but could be paresthesias on one side of the body or speech arrest [4].

Patients with episodic migraine can remit, remain unchanged, or progress to high-frequency episodic or chronic migraine over time. Chronic migraine is associated with a substantially greater personal and societal burden, an increased number of comorbidities, and patients may develop progressive brain abnormalities [5–7]. Migraine is a serious and widespread health problem as measured by years lived with disability (YLDs) and is considered the sixth highest cause of disability worldwide, while medication overuse headaches follow at eighteenth [8]. By adding these two conditions together, headache becomes the third most common cause of disability measured in YLDs worldwide [9].

All migraineurs require acute care treatment, and up to 40 % of episodic migraineurs could benefit from preventive therapy; but few undertake it. Poor quality of life is one of the various appropriate reasons to start a patient on migraine preventive therapy. All patients with chronic migraine should be offered prevention to attempt to reduce the number and severity of their headache days. Currently, oral pharmaceutical agents are recommended as the first choice for preventive migraine treatment worldwide and invasive treatments are suggested only occasionally [10–12].

The main goals of migraine preventive treatment include: reducing headache frequency, severity and intensity; restoring function; and preventing progression to chronic migraine. There is evidence that valproate, topiramate, metoprolol, propranolol, timolol and flunarizine are effective for episodic migraine prevention and should be offered to appropriate migraineurs to reduce migraine attack frequency and severity (level A medications) [10]. Frovatriptan (primarily indicated and usually used for acute care of migraine) is effective for prevention of menstrual migraine (level A), while lamotrigine is ineffective for migraine prevention and should not be used (level A) [10–12]. Although commonly used in clinical practice, amitriptyline does not carry adequate evidence for migraine prevention (only class II studies) [10]. For prevention of chronic migraine (with and without medication overuse) only one treatment is approved in the US by the Food and Drug Administration (FDA) and that is onabotulinumtoxinA. Topiramate studies do show efficacy but there is no approval for this condition [13–15]. Additionally, a variety of behavioral medicine treatments, such as biofeedback training and cognitive restructuring, are available for these patients [16].

### Do we need novel migraine preventive treatments?

Although there are many approved and unapproved preventive treatments for migraine, they are often insufficient to manage migraineurs effectively, even in the right hands. There are issues of efficacy, tolerance, safety, adherence, pharmacophobia and nocebo response, all suggesting the need for better treatments. There are several outcome measures used in clinical trials to qualify the efficacy of migraine prevention. The most common is a decrease in migraine or headache days per month compared to baseline, and the proportion of responders to the treatment, defined as those patients that report more than a 50 % decrease in migraine days per month after a given treatment (the 50 % responder rate). The number needed to treat (NNT; defined as the number of patients who need a specific treatment to prevent one additional bad outcome, e.g. a migraine attack) for responders varies from 4–6 in several randomized trials for migraine prevention (indicating that 4–6 patients suffering from migraine must be treated in order for one to reach a 50 % decrease in migraine days per month). The decrease in migraine days per month after extracting the placebo effect varies from 1.2–1.8 [17, 18]. This absolute improvement, not including the placebo effect that indeed exists and improves treatment outcomes in real life, looks very small; there is clearly room for improvement. There is also evidence that migraineurs are very sensitive to adverse events (AEs) of preventive medications and more likely to withdraw from treatment because of AEs in comparison to epileptics, as one meta-analytic study with topiramate showed [19]. Generally, one out of five patients treated with any migraine preventive pharmaceutical agent will discontinue treatment because of tolerability and safety reasons [20], as did one out of twenty patients treated with placebo in randomized controlled studies [21]. In this context, a medication’s safety profile matters significantly to migraineurs and impacts adherence considerably [22].

Adherence is poor in migraine preventive treatments, as in most conditions requiring chronic therapy. Only one out of four patients complies with treatment in chronic migraine when it is required for 6 months, and this decreases to one in five when treatment duration increases to 1 year. Adherence is related to drug tolerability and efficacy [23], once again indicating the need for novel and better anti-migraine therapies.

Pharmacophobia refers to the fear of medication. Taken together with the nocebo effect, which refers to the experience of AEs related to patients’ negative expectation that a treatment will most likely harm instead of help, these two concepts control treatment adherence and outcome in migraine and other headaches significantly. It is known that one out of twenty headache sufferers discontinues treatment because of the nocebo effect [22]. For all of these reasons, improved therapeutic approaches, including non-pharmaceutical ones, should continue to be researched.

### Novel preventive anti-migraine treatments and interventions

In the last 10 years, several new acute care and preventive migraine treatments have surfaced, some of them related to the calcitonin gene-related peptide (CGRP). The small molecule CGRP receptor antagonists have been effective, without evidence of vasoconstriction in animals, but there have been issues of safety, thus far preventing further development. In the last few years we have seen the development of four monoclonal antibodies targeting CGRP or its receptor (CGRP-mAbs). Phase 2 trials show promising efficacy data with limited adverse events and almost no serious adverse events.

### Monoclonal antibodies to CGRP ligand and receptor

Although the small molecule agents that target the CGRP receptor are still under investigation, the recent development of humanized antibodies to CGRP and its receptor appear more promising for three important reasons: they are unlikely to cause liver toxicity or other serious AEs; they are biological products with extreme specificity for their target and very long half-lives, compared to oral medications; and they may have considerably better tolerability and safety profiles [24].

To demonstrate that CGRP-mAbs will be useful in migraine treatment, four such antibodies have been tested successfully in animal models and are currently in phase 3 trials in the US [25–27]. The major concern is that blocking CGRP, a potent and ubiquitous vasodilator, may cause cardiovascular effects, including medication-induced hypertension, interactions with the efficacy of anti-hypertensive drugs and inhibition of ischemia-related coronary vasodilatation [28]. Monoclonal antibodies may cause biological effects within other organ systems. Infusion and immunological reactions are also potential adverse events [29]. Another important concern is the long half-lives of mAbs, which prevent immediate clearance in case of severe AEs [24]. Of course, this is also one of its major benefits, as long half-lives prevent the need for frequent dosing. Lastly, the possible development of neutralizing anti-drug antibodies may abolish the effectiveness of the treatment, but this has not been a significant issue thus far in the trials. To eliminate these concerns, several phase 1 studies have been conducted in humans for each of these therapies and show that sustained CGRP inhibition is not associated with hemodynamic or ECG changes, nor have other significant safety concerns emerged [30, 31].

Four humanized, monoclonal antibodies are currently in phase 3 trials for the prevention of episodic or chronic migraine and even cluster headache: ALD403 (Alder Biopharmaceutical, Bothell, WA, USA); LY2951742 (Eli Lilly, Indianapolis, IN, USA); AMG 334 (Amgen, Cambridge, UK); and TEV-48125 (Teva, Petah Tikva, Israel) [32–36] The principal findings of these phase 2 studies are summarized in Table 1. Published data are available for three antibodies: LY2951742; ALD403; and TEV-48125 [32, 33, 35, 36]. Data for AMG 334 is based on a presentation at the International Headache Society Congress (IHC) in Valencia, in May 2015 [34]. Some CGRP antibodies have been tested in patients suffering from episodic migraine, others in a wider range of headache frequencies, from high frequency episodic migraine to chronic migraine (Table 1). The primary endpoint was common in all studies of episodic migraine (change in migraine days per month from baseline). TEV-48125 was also tested in chronic migraine using a novel primary endpoint of decrease in the total number of hours per month of headache at 3 months versus baseline [36]. The different frequencies of migraine in these studies, the different types of migraine and the different primary endpoints make it difficult to adequately compare these treatments.

**Table 1:** Monoclonal antibodies target to CGRP pathway in clinical trials phase II for migraine prevention

Antibody	Target	Study size population (active vs. placebo)	Inclusion criteria	Mean baseline MHD/28d (active vs. placebo)	Treatment duration (weeks)	Dose, root & frequency	Primary outcome (change from baseline)	Active vs. placebo change of primary outcome	50 % responder rate (NNT)	Dropout ratio	Common AEs	Anti-drug antibodies
LY2951742 [33]	CGRP	218 (108 vs. 110)	Episodic migraine (4–14 MHD/28d)	6.7 vs. 7.0	12	150 mg sc; every 2 weeks	MHD/28d (at 9–12 weeks)	−4.2 vs. -3.0 (1.2 days difference, p = 0.003)	70 % vs. 45 % (4.0)	12 %	Erythema; Site pain; infection; abdominal pain	18.7 %
ALD403 [32]	CGRP	163 (81 vs. 82)	Episodic migraine (5–14 MHD/28d)	8.4 vs. 8.8	12	1 g iv; once	MHD/28d (at 5–8 weeks)	−5.6 vs. -4.6 (1 day difference, p = 0.03)	75 % vs. 54 % (4.7)	6.2 %	Tooth abscess; dizziness; ECG changes; dry mouth	14 %
TEV48125 [35]	CGRP	297 (96, 97 vs. 104)	Episodic migraine (8–14 MHD/28d)	11.4 vs. 11.5	12	225 & 675 mg sc; every 4 weeks	MHD/28d	2.64 days difference, p < 0.001	59 % vs. 28 % (3.2)	9.1 %	Injection site discomfort; redness	1 %
TEV48125 [36]	CGRP	264 (175 vs. 89, 3 arms)	Chronic Migraine	16.4 vs. 16.8 (157.7 vs. 169.1 hours/mo)	12	225/675 & 900 mg sc; every 4 weeks	HH/28d	−67.5 vs. -37.1 (30.4 hrs difference, p = 0.001)	NA	14.4 %	Injection site; pruritus	1 %
AMG334 [34]	CGRP receptor	483 (4 arms)	Episodic migraine	8.7	12	7, 21 & 70 mg sc; every 4 weeks	MHD/28d (at 9–12 weeks)	−3.4 vs. -2.28 (1.1 day difference, p = 0.021)	47 % vs. 30 % (5.9)	NA	Fatigue; influenza; nasopharyngiitis; arthralgia; back pain	NA

TEV48125: efficacy results for the higher dose used (675 mg in episodic migraine and 900 mg in chronic migraine) [35, 36]; AMG344: efficacy results for the dose of 70 mg [34]; MHD: Migraine Headache Days; HH: Headache Hours; NNT: number needed to treat

All four episodic migraine studies show the monoclonal antibodies starting to work in 4 weeks versus placebo. The TEV-48125 study shows significance over placebo in less than 1 week [36]. In Table 2, a comparison between CGRP monoclonal antibodies and currently available oral treatments in the prevention of episodic migraine [37] is presented. NNT are comparable. However, these comparisons merely present a rough idea of comparability. Since each trial is different and there are no head-to-head studies, no conclusions can be drawn. Numbers needed to harm (NNH) appear to favor TEV-48125, ALD403 and LY2951742. NNTs for treatment discontinuation due to AEs and relative risks for AEs favor LY2951742, ALD403 and TEV-48125 as well [37]. Adverse events specific to CGRP actions (e.g. hypertension and coronary vasodilatation) have not been an issue in these trials. Lastly, the development of anti-CGRP antibodies remains to be tested in long-term follow-up studies. In the Teva trials, a few patients had neutralizing antibodies even before starting the trial, the number did not increase after the trial and no significant problems developed in those patients. The other trials had patients with more neutralizing auto-antibodies with apparently no deleterious effects. Results from phase 2 studies in episodic migraine showed that patients treated with either ALD403 or LY2951742 developed anti-CGRP222 antibodies within 24 weeks (14 % and 18 %, respectively) [32, 33], indicating that some will develop neutralizing auto-antibodies (NAbs). Experience from biological agents used in multiple sclerosis (MS) shows that about 15 % of patients develop neutralizing auto-antibodies, which decreases biological activity and consequently their therapeutic action [38].

**Table 2:** Comparison between CGRP monoclonal antibodies, transcutaneous supraorbital nerve stimulation and current available oral treatments in the prevention of episodic migraine

Drug	LY2951742 [33]	ALD403 [32]	TEV48125 [35]	AMG334 [34]	tSNS [42]	Valproate [37]	Topiramate [37]	Propranolol [37]	Amitriptyline [37]
NNT	4	4.7	3.2	5.9	3.8	4	3	4	4
NNH	20	20	∞	NA	∞	7-14	2-17	NA	NA
Relative risk for AE	1.07	1.09	1	NA	1	1.2	1.8	2.1	1.9
NNT for discontinuation due to AE	∞	∞	∞	NA	∞	NA	16	16	19

tSNS: transcutaneous supraorbital nerve stimulation; NNT: number needed to treat; NNH: number needed to harm; AE: adverse event; NA: non applicable; ∞: NNH, same percentage of patients experienced any adverse event in both placebo and drug treated patients; NNTs for discontinuation due to adverse events, no patient treated with the drug discontinued because of adverse event

Adherence to treatment is a critical factor in migraine management, but is often underuestimated [23, 39, 40]. Many patients who begin migraine prevention with oral agents no longer take these medications 3–6 months after they start them. One study shows that 73.4 %, 70.2 % and 67.6 % of 4,634 migraineurs who initiated migraine prevention with antidepressants, anti-epileptics and beta-blockers, respectively, were found non-adherent 6 months later [40]. More than a few factors power adherence, including tolerability and frequency of treatment administration, and both favor CGRP-mAbs; but this has to be proven in long-term follow-up studies.

Overall, CGRP-mAbs look like promising options for migraine and chronic migraine prevention with impressive responder rates, improved safety and tolerability, absence of liver toxicity and long half-lives leading to infrequent dosing. Depending on the results of phase 3 trials, these therapies could become first-line in episodic and chronic migraine prevention. No doubt they will be costly, but if they are effective, prevent disability and frequent visits for emergency care, they may be cost-effective.

Notably, oral CGRP receptor antagonists are still under investigation for acute care and prevention of migraine, with promising results. Telcagepant did not show efficacy over placebo in women suffering from perimenstrual migraine [41], but in another placebo-controlled trial for migraine prevention, telcagepant resulted in a larger reduction from baseline than placebo for mean monthly headache days (month 1: 140 mg, −2.9; 280 mg, −3.1; placebo, −1.7; *P* <0.05) and migraine/probable migraine days (month 1: 140 mg, −2.7; 280 mg, −3.0; placebo, −1.6; *P* <0.05) [42]. In both studies elevation of serum alanine aminotransferase was observed in a proportion of patients (2.5 %, when the drug is taken daily), indicating safety concerns.

### Neurostimulation

Invasive and non-invasive central or peripheral neurostimulation techniques have been developed by different companies with encouraging results for various headache disorders, including migraine and cluster headache. Recently the Neuromodulation Appropriateness Consensus Committee concluded that extracranial nerve stimulation should be considered in the algorithmic treatment of migraine [43]. To date there is evidence that only two non-invasive techniques are effective in migraine prevention: transcutaneous supraorbital and supratrochlear nerve stimulation (tSNS) and vagus nerve stimulation (VNS). Invasive techniques are also under investigation, yet they target non-responders to the currently available therapies in chronic migraine and in chronic cluster headache; but will not discussed here, even though they appear effective in early trials [44].

#### Transcutaneous supratrochlear nerve stimulation (tSNS)

The efficacy and safety of tSNS for prevention of episodic migraine has been evaluated in a randomized, double-blind, sham-controlled trial published in *Neurology* [45]. Sixty-seven patients were treated with daily tSNS or sham sessions for 20 minutes per day for 3 months. The change in migraine days per month from baseline was significantly better in tSNS patients than in sham-treated patients (−2.06, *P* = 0.023 vs. −0.32, *P* = 0.608) and had a 50 % responder rate (38.1 % vs. 12.1 %, *P* = 0.023, NNT = 3.8). Importantly, the primary endpoint of change in migraine days per month just missed significance in the intention-to-treat group (*P* = 0.054) and was not significantly different in per protocol analysis (*P* = 0.06) in the comparison between groups (tSNS and sham-treated). Rescue migraine medication intake was significantly reduced in the verum but not in the sham group. Notably, patients did not report many AEs [45]. It should be noted that it was difficult to completely blind this study. Although not reported in the study, personal reports from several patients using the device indicated that the paresthesias were strong enough to cause unblinding and discontinuation from the trial. In an observational survey among 2,313 patients, 54.4 % were satisfied with the treatment. Only 4.3 % of individuals reported one or more AEs, such as local pain/intolerance to paresthesia (2.03 %), arousal changes (0.82 %) and headache after the stimulation (0.52 %). A transient local skin allergy was seen only in 0.09 % [46]. In a second uncontrolled, observational study of patients suffering from episodic migraine without aura, a 2-month treatment with Cefaly (Cefaly Technology, Grâce-Hollogne, Belgium) significantly reduced migraine days per month compared to the baseline period, without reported AEs [47].

#### Transcutaneous vagal nerve stimulation (tVNS)

In a randomized, sham-controlled pilot study for prevention of chronic migraine with transcutaneous vagal nerve stimulation (tVNS) using the gammaCore stimulator (three daily 90-second stimulations for 2 months) the responder ratio was 15 % (4 out of 26) in the verum group compared to zero (none out of 23) in the sham-stimulated group (NNT = 6.7) [48]. This beneficial effect was confirmed in the subsequent open-label phase [49]. Besides neck muscle (platysma) contractions in some patients, there were no significant AEs. Apart from these preliminary data, further studies are needed to determine the role of tVNS in migraine prevention. Recent data indicate good efficacy for the symptomatic treatment of both migraine and cluster headache, and also for the prevention of cluster headache [50, 51].

#### Sphenopalatine ganglion stimulation

Sphenopalatine ganglion stimulation is under investigation for the symptomatic and preventive treatment of both migraine and cluster headache. When used acutely for attacks in patients with chronic cluster headache, many attacks significantly improve within 15 minutes, which was the primary endpoint. Some patients also experience a decrease in frequency of attacks over time. The device is available in Europe and has a CE mark (a CE mark is a logo that is placed on medical devices to show they conform to the requirements of the Medical Device Directive; it shows that the device is fit for its intended purpose as stated and meets legislation relating to safety; and it shows the product can be freely marketed anywhere in the European Union without further control). It is more invasive than tVNS, since implantation of the device through the oral cavity under general anesthesia is required [52].

#### Transcranial magnetic stimulation

There are data indicating that high-rate, repetitive transcranial magnetic stimulation is effective in migraine prevention [53], but further investigation is needed.

In general, these preliminary data of non-invasive neurostimulation in migraine prevention and symptomatic treatment of attacks in chronic cluster headache point towards a safety advantage, but whether they will be efficacious enough is yet to be determined. Additionally, neurostimulation may be a much better treatment option for migraineurs that do not stay on oral medications due to a variety of factors, such as pharmacophobia and nocebo behaviors. There are many studies of invasive implanted stimulators for chronic migraine and other headaches that are not discussed here.

## Conclusions

Migraine is a common, chronic neurovascular disorder of the brain with cranial autonomic findings. It is characterized by recurrent, severe attacks of headaches often associated with other symptoms and much disability, as well as personal, familial and societal impact. Currently, there only are a limited number of preventive treatments for migraine worldwide, and they often are ineffective and cause AEs leading to low retention rates. Better therapies are badly needed. CGRP-mAbs to the ligand and receptor display at least, if not greater, comparable efficacy to the currently available oral therapies, with better safety data and perhaps adherence in phase 2 trials. Neurostimulation also appears promising, despite the limited evidence. Both monoclonal antibodies and neurostimulation appear to offer effective, novel management for migraine prevention and acute care of chronic cluster headache. We look forward to further results of these above therapies.
